# Nervous Diseases and Modern Life

**Published:** 1895-03-02

**Authors:** 


### Nervous Diseases and Modern Life.

In the *Contemporary* for last month Professor Clifford Allbutt repeats his attack on the prevalent idea that modern life is productive of decadence of race, especially in regard to its influence on the nervous system. He maintains that nervous diseases are not increasing; that work no more injures nerves than it does muscles; that, so far from over-excitability of nerves being a disease, excitability is their proper function, and the more excitable they are the better; and that over-education and over-pressure of boys and girls is "fudge."

Within his limitations we may, perhaps, agree with him, but it is important to recognise what these limitations are. First of all, he ties down the question to mere nervous tissue, and if we consider how narrow are the bounds of our knowledge of diseases of nerve filaments and cells, we may admit that it would be difficult to either prove or disprove either their waning or their growth. He says, "When disease of the skull invades the underlying brain, the resulting disorders are not to be regarded as primarily nervous. So, again, if the brain suffer, as very often it does, from disease of the blood vessels which should feed it and do not, we ought not to regard the consequent disorders as nervous in nature. In like manner I might proceed to show that a very large number of diseases called nervous are not primarily diseases of nervous tissue, but, beginning near these, engage them in a secondary way. Or certain specific affections of the nervous tissues may be secondary to intoxications, such as lead, alcohol, diphtheria, influenza, and so forth; and in such cases we must again acquit the nervous system of primary defect." Having thus cut away the great mass of what are commonly called nervous diseases on the ground that they are only nervous in a secondary sense, he proceeds to narrow down the influence of modern life in their production in an equally ruthless way, cutting adrift, as not germane to the question, much which is the very essence and peculiarity of modern life. The hurry, the excitement, the want of rest, the neglect of food, the champagne, the tea, the stuffy air, the meretricious entertainments, are put aside as not touching the question, and finally he saves himself by a footnote which says, "The main subject of this essay is the relation of work to the nervous system; distress and anxiety are factors rather of alimentary and cardio-renal changes than of nervous disease." Within these limitations it is easy to agree with him; but then the peculiarity of modern life is not the amount of work that is done, but the conditions amid which it is performed. It is a mere platitude to say that "the men or women who, having inherited a fairly stable nervous system, work their brains so as to get the most out of them, are temperate in meat and drink, and secure their own portion of fresh air; who

> Arm their constant and their nobler parts
> Against giddy, loose suggestions, and who keep out of railway accidents, may fight their way without making for the doctor." Is this a true picture of the characteristics of modern life? When the sort of people who read magazines think of nervous diseases they do not draw fine pathological distinctions, but include all those diseases which show themselves by altered nervous action, together with all those diseases of other organs which are brought on by nervous worry.

In this sense we think it would be difficult to prove that the hurry and anxiety, the very "fulness" of modern life which to some is its chief charm, are not provocative of neuroses, and even of organic nerve disease. Professor Allbutt delights greatly in the contemplation of the splendid specimens of humanity which the freer life of the middle classes at this end of the century has produced, but those who work in cities, and watch on the one side the product of the slums, and on the other the victims of the office, cannot fail to see that the conditions which produce the splendid specimens, viz., the free life, the open air, the plentiful diet, are not the lot of the majority, and that large numbers of people have to work under conditions both of strain and insalubrity by which their nervous health becomes impaired. It is interesting no doubt to be told that mere intellectual effort does no harm to the nervous system, and that such work can by no means produce disease; but it is not by mere intellectual work that livings are now earned or positions are attained. The long hours and constant anxiety, the excitement of business or of politics when all is trembling in the balance, the sleepless nights through which the brain goes on revolving in the track set for it by day, the indigestion and neglected meals, the sedentary life interspersed with wild bursts of frantic hurry, the double life of business work and social struggle, these are the concomitants of the brain work of the present day, and although they do not come within the category of the proper and orderly use of the mind which Professor Allbutt seems to have in his eye, and although when they do harm to the nervous system they often do it indirectly, as he would say by way of "alimentary and cardio-renal changes," still, that hardly touches the proposition which so many will be found ready to maintain, that brain work, as work is so often now done, and modern life, as life is often now led, are conducive, indirectly perhaps, but still conducive to nervous diseases.

So far we have chiefly had in mind diseases which manifest themselves by disorders of the nervous system. There is, however, another aspect of the question which cannot be entirely ignored. No one knows better than Professor Allbutt—in fact, few have done so much to popularise the idea—that many disturbances of function, commonly regarded as local diseases, are really due to perverted innervation. Years ago he turned his batteries upon the gynecologists, showing that they were using couches, cauteries, and mechanical contrivances for what were pure neuroses and demanded rides on horseback for their cure. He did the same for the dyspeptics, showing that they often wanted quinine and arsenic and hydropathy rather than the bismuth and the rhubarb which were their more common fate. Adding to these the gout, and possibly the Bright's disease, caused by worry and anxiety, we have a whole group of maladies which, although not perhaps entirely diseases of the nerves, are trophic disorders set up by perverted or inhibited nerve supply. As Professor Allbutt propounds his somewhat neat and narrow thesis we agree with him; but there is a larger question, and that we think requires a different answer.

